# Periodontal indexes of two types of 3 x 3 retainers: 0.032-in SS V-loop *versus* 0.0215-in SS coaxial - a randomized crossover trial

**DOI:** 10.1590/2177-6709.28.6.e2323175.oar

**Published:** 2024-01-08

**Authors:** Diogo Marques SAPATA, Cléverson de OLIVEIRA E SILVA, Renata Corrêa PASCOTTO, Thais Maria Freire Fernandes POLETI, Maristela Sayuri Inoue ARAI, Adilson Luiz RAMOS

**Affiliations:** 1 Universidade Estadual de Maringá, Departamento de Odontologia (Maringá/PR, Brazil).; 2 Universidade Norte do Paraná, Departamento de Odontologia (Londrina/PR, Brazil).; 3 Tokyo Medical and Dental University, Maxillofacial Department (Tokyo, Japan).

**Keywords:** Dental plaque, Orthodontic treatment, Retainer, Periodontal index

## Abstract

**Objective:**

This randomized crossover trial evaluated periodontal indexes of two types of 3 x 3 retainers (a modified 0.032-in SS V-loop retainer and a conventional 0.0215-in SS coaxial wire retainer) after bonded for six months. Also, bonded failure rate, and a questionnaire about comfort, ease of cleaning and overall preference were recorded.

**Material and Methods:**

15 patients were enrolled in this study who used both retainers for six months each, having a 15-day wash-out interval between each bonded retainer usage. The following periodontal index were recorded: Plaque Index (PI), Calculus Index (CI) and Gingival Index (GI). Patients answered a questionnaire to assess comfort, ease of cleaning and overall retainer-type preference. Rate of bonding failure was also evaluated.

**Results:**

V-Loop retainer showed higher PI (P<0.05) as compared to conventional 0.0215-in coaxial wire retainer. However, CI and GI presented no statistically significant differences between both types of retainers. The conventional 0.0215-in coaxial wire retainer was chosen as the most comfortable (*p*<0.05), although no statistically significant differences were found for all other questionnaire answers. Bonding failure events were more observed in the 3x3 V-Loop retainer (*p*<0.002), as compared to the conventional 0.0215-in coaxial retainer.

**Conclusion:**

V-Loop retainer showed higher PI (*p*<0.05), higher bonding failure rate and less comfortable, as compared to conventional 0.0215-in coaxial wire.

## RESUMO

**Objetivo:**

Este estudo cruzado e randomizado avaliou os índices periodontais de dois tipos de contenções 3x3 (uma contenção aço V-Loop de 0,032” modificada e uma contenção convencional de fio coaxial aço de 0,0215”) após colagem, por seis meses. Além disso, foram registradas a taxa de falha na colagem e um questionário sobre conforto, facilidade de limpeza e preferência geral.

**Material e Métodos:**

Foram incluídos nesse estudo 15 pacientes que usaram ambas as contenções por seis meses cada, com intervalo de quinze dias entre cada contenção fixa. Foram registrados os seguintes índices periodontais: Índice de Placa (IP), Índice de Cálculo (IC) e Índice Gengival (IG). Os pacientes responderam a um questionário para avaliar o conforto, a facilidade de limpeza e a preferência geral pelo tipo de contenção. A taxa de falha de colagem também foi avaliada.

**Resultados:**

A contenção V-Loop apresentou maior IP (*p*<0,05) em comparação ao fio coaxial convencional. Entretanto, IC e IG não apresentaram diferenças estatisticamente significativas entre as contenções testadas. A contenção convencional de fio coaxial 0,0215” foi escolhida como a mais confortável (*p*<0,05), embora não tenham sido encontradas diferenças estatisticamente significativas para todas as outras respostas do questionário. Eventos de falha de colagem foram mais observados na contenção V-Loop 3 x 3 (*p*<0,002) em comparação com a contenção coaxial convencional de 0,0215”.

**Conclusão:**

A contenção V-Loop apresentou maior IP (*p*<0,05), maior taxa de falha de colagem e foi menos confortável em comparação ao fio coaxial convencional 0,0215”.

## INTRODUCTION

There is a consensus that orthodontic retainers must be used after completing orthodontic treatment, to avoid relapse and aging effect over dental arches.^1^ ^-^ ^6^ Vacuum-formed or Hawley appliances are the most common options for the upper arch, and bonded wires contoured from canine to canine are the most recommended choices for the lower arch. 

Long term periodontal health is a concern when fixed retainers are chosen. Some authors describe some negative impact on lower anterior teeth periodontium due to those fixed retainers, while others did not find any differences among those with or without them, in a long-term perspective.^6^ ^-^ ^15^ Besides such controversial points, there is a consensus that fixed retainers should be used to avoid malalignment specially for the lower arch. 

Thus, such retainers should be comfortable and easy to be cleaned, helping periodontal health with time. In 2015, a 3x3 V-loop retainer was suggested, presenting a design with V-formed curves perpendicular to the tooth axis to be easy to clean, as well as overall stability properties of retainers.^8^ ^,^ ^9^ However, no study tested such characteristic on periodontal condition with time. 

Thus, the aim of this study was to evaluate periodontal indexes of two types of 3 x 3 retainers (a modified 0.032-in SS V-loop retainer and a conventional 0.0215-in SS coaxial wire retainer). The null hypothesis tested was that both retainers would present similar periodontal performance. 

## MATERIAL AND METHODS

This randomized crossover trial was approved by the Ethics Research Committee of the State University of Maringá (CAEE: 12854719.1.0000.0104). The patients signed a free and informed consent form, in accordance with the Guidelines and Regulatory Standards of the National Health Council (Resolution No. 196/96), following the CONSORT 2010 statement.^16^

The sample calculation was performed considering the primary variable PI, using G*Power 3.1 software. For repeated measurement tests between factors, with sample effect equal to 1, beta value 0.95 and alfa 0.5, considering two groups, two measurements, the resulting sample size was 14. However, based on previous studies, 19 patients were recruited, considering a drop out of 20%.^17^ ^,^ ^18^

**Figure 1:**
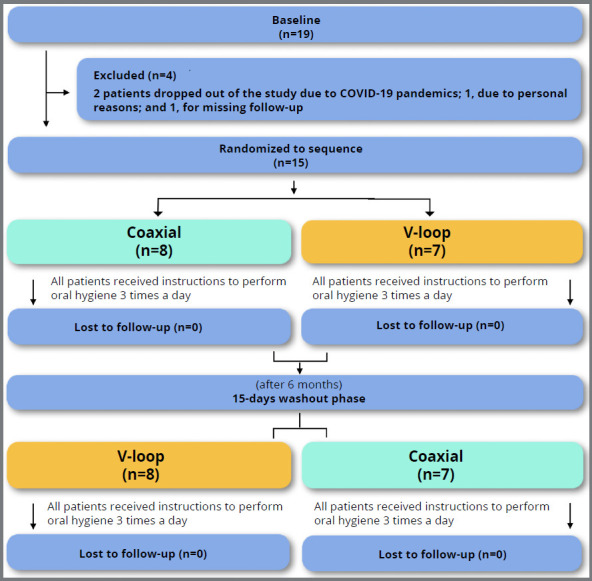
Diagram of the experimental protocol.

Inclusion criteria were as follows: good periodontal health condition, no-smoking habit, no systemic disease, well-aligned lower anterior teeth or maximum of 2-mm irregularity, right-handed and aged 18 to 30 years-old. Exclusion criteria were as follows: undergoing orthodontic treatment at the time or presenting any coordination disability. This experimental protocol was based on previous studies.^17^ ^,^ ^18^ The patients were given randomly generated envelopes. This was a crossover study, presenting the following phases in the experimental design (Fig 1):

- *Baseline* - All individuals (n=19) underwent scaling, and dental prophylaxis was performed 15 days prior to the use of retainers. On day zero, periodontal parameters were normal.
- After retainers confection, 9 patients used the 0.0215” coaxial retainers and 8 used the V-loop retainer for six months. Periodontal parameters were recorded at the end.
- *Washout* - With drop out equivalent to 4 patients, this phase was reached with 15 patients. All retainers were removed, along with the removal of residual resin, in addition to polishing, and a 15-day interval was used for normalization of the parameters.
- A new retainer was bonded, as follows: Patientes who had used the V-loop type started using the coaxial retainer, and vice versa, for another six months. Periodontal parameters were recorded at the end.

All patients received instructions to perform oral hygiene three times a day, and received a hygiene kit comprising a toothbrush (Colgate^®^ SlimSoft™ Black, Colgate-Palmolive, São Bernardo do Campo-SP, Brazil), toothpaste (Colgate^®^ Total 12 Clean Mint, Colgate-Palmolive, São Bernardo do Campo/SP, Brazil) and dental floss (MedFio^®^, Medfio Indústria e Comércio de Artigos Odontológicos - EirelI, Pinhais/PR, Brazil). Two hygiene kits were given to the patients, one for each retainer test interval. They were instructed to use the Bass method^19^, which involves holding the toothbrush at an angle so that the bristles point at the gum line and making short back-and-forth strokes, followed by sweeping the brush from under the gum toward the edge of the tooth.

All the periodontal evaluations were performed by the same operator. The calibration of the evaluator was submitted to the Kappa agreement test for 20% of the sample, and the result obtained was 0.91 (*p*<0.05).

At the end of the twelve months, the patients answered a questionnaire to evaluate both retainers regarding comfort, easiness to hygiene, the necessity of a floss guide and their overall preferred retainer type. 

**Figure 2:**
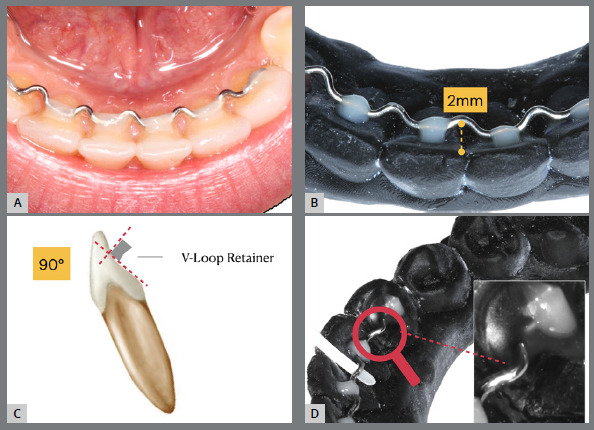
V-loop retainer illustration: A) in mouth; B) occlusal view; C) lateral view; D) lingual view.

The V-loop retainer was made by a single operator on the plaster models. The V-loop retainer was made with a 0.6-mm stainless steel orthodontic wire (Morelli, Sorocaba, Brazil) respecting the model with “v” bends moving away from the proximal surface of the tooth by 2mm and forming a curve perpendicular to the long axis of the tooth (Fig 2). The conventional 0.0215-in SS coaxial retainer (Orthometric, Marília/SP, Brazil) was contoured for the six lower anterior teeth. All retainers were bonded by using the same resin composite (Resin Z100TM 4g - 3M, Joinville/SC, Brazil), performed by a single operator. A OneGloss PS tip drill (Shofu, São Paulo/SP, Brazil) was used for polishing. 

### PERIODONTAL EVALUATION

**Figure 3:**
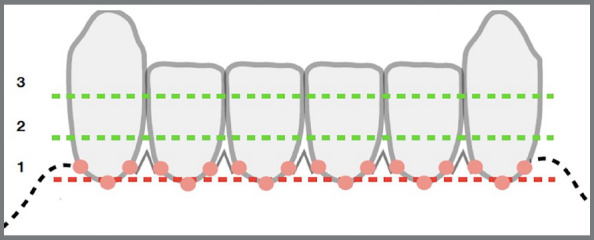
Diagram used for periodontal measurements recording.

Periodontal parameters were evaluated for three areas of the anterior lower teeth: mesiolingual, distolingual, and lingual. Dental plaque index (PI),^20^ dental calculus index (CI),^21^ and gingival index (GI)^22^ were recorded. PI evaluation followed the simplified Silness and Löe method.^20^ Each tooth was divided into three surfaces (mesiolingual, distolingual and lingual), and then separated by the lower, middle, and upper thirds. The plaque was evidenced with 1% toluidine blue dissolved in distilled water. Scores from 0 to 3 were recorded: 0 = absence of plaque; 1 = presence of plaque on at least 1/3 of the tooth surface, 2 = presence of plaque in 2/3 of the tooth surface, and 3 = presence of plaque in 3/3 of the tooth surface. Then, the median for each surface was calculated (Fig 3). 

Calculus Index (CI), as described by Ramfjord^21^, was verified with a North Carolina single tip probe #15 (Hu-Friedy Chicago, Illinois, USA) inserted to the three surfaces (lingual, mesiolingual, and distolingual). The presence of dental calculus was identified when the probe stuck, in addition to a visual verification. Scores 0 or 1 were recorded: 0 = absence of calculus, 1 = presence of calculus. 

Löe’s gingival index (GI)^22^ was evaluated using a North Carolina single tip #15 probe (Hu-Friedy Chicago, Illinois/USA). Score 0 was recorded for healthy gingiva; and score 1, for the presence of bleeding in the gingival margin. A diagram was used to record all indexes (Fig 3).

The bonding failure rate were also recorded for each retainer, being considered as a dichotomous variable in which the absence of composite resin on the lingual surface of mandibular anterior teeth indicated failure. 

### STATISTICAL ANALYSIS

Fisher test was used for PI comparisons; and McNemar test, for CI, GI and for the questionnaire. All the tests considered a significance level of 5%. 

## RESULTS

From the initial 19 patients, 4 dropped out of the study: 1 for personal reasons, 2 because of the COVID-19 pandemic, and 1 for not attending a follow-up appointment (Fig 1). The baseline demographic characteristics were: 4 men and 15 women, with a mean age of 23.8 years, and standard deviation of 3.76.

**Figure 4:**
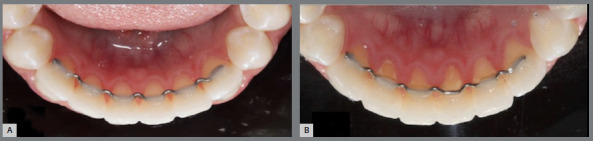
V-loop retainer at baseline (A) and after 6 months (B).

**Figure 5:**
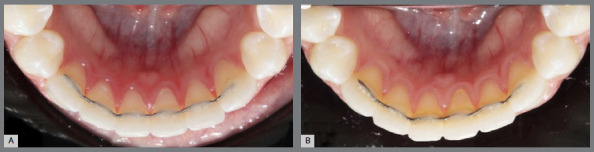
Coaxial retainer at baseline (A) and after 6 months (B).

**Figure 6:**
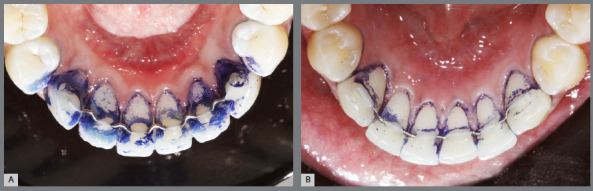
Plaque recording for V-loop retainer (A) and coaxial retainer (B).

Table 1 shows the frequency and percentage of PI, per dental surface for both groups. PI was slightly higher (*p*<0.01) for V-loop retainers in all evaluated surfaces (mesiolingual = 57.8%, distolingual = 56.7% and lingual = 57.8%) (Figs 4, 5 and 6).

**Table 1:** Frequency and Plaque Index scores for V-loop and coaxial retainers.

Plaque Index	V-Loop n (%)	Coaxial n (%)	p -value
Mesiolingual			< 0.001
0	6 (6.7)	0 (0.0)	
1	10 (11.1)	18 (20.0)	
2	22 (24.4)	28 (31.1)	
3	52 (57.8)	44 (48.9)	
Distolingual			0.005
0	7 (7.8)	0 (0.0)	
1	10 (11.1)	18 (20.0)	
2	22 (24.4)	28 (31.1)	
3	51 (56.7)	44 (48.9)	
Lingual			0.001
0	3 (3.3)	0 (0.0)	
1	13 (14.4)	18 (20.0)	
2	22 (24.4)	28 (31.1)	
3	52 (57,8)	44 (48.9)	

*Scores: 0 = no plaque; 1 = 1/3 plaque; 2 = 2/3 plaque; 3 = 3/3 plaque.

Tables 2 and 3 show the frequency and percentage for dental CI and GI. There were no statistically significant differences between the retainers. 

**Table 2:** Frequency and Calculus Index scores for V-loop and coaxial retainers.

Calculus Index	V-Loop n (%)	Coaxial n (%)	p -value
Mesiolingual			0.715
0	43 (47.3)	45 (50.0)	
1	47 (52.7)	45 (50.0)	
Distolingual			0.223
0	51 (56.6)	58 (64.4)	
1	39 (43.4)	32 (35.6)	
Lingual			0.394
0	69 (76.6)	65 (72.2)	
1	21 (23,4)	25 (27.8)	

*Scores: 0 = absence of calculus; 1 = presence of calculus.

**Table 3:** Frequency and Gingival Index scores for V-loop and coaxial retainers.

Gingival Index	V-Loop n (%)	Coaxial n (%)	p -value
Mesiolingual			0.763
0	45 (50.0)	43 (47.3)	
1	45 (50.0)	47 (52.7)	
Distolingual			0.069
0	36 (40.0)	49 (54.4)	
1	54 (60.0)	41 (45.6)	
Lingual			0.180
0	57 (63.3)	66 (73.3)	
1	33 (36.7)	24 (26.7)	

* Scores: 0 = healthy periodontium; 1 = presence of bleeding at the gingival margin.

Questionnaire results are presented in Table 4, where 87.7% of the patients opted for coaxial retainers regarding comfort (*p*< 0.05). On the other hand, 60% reported that the V-loop retainers were better to be cleaned, but without statistical significance. Regarding the need for the floss threader, no clinically difference were registered. Considering overall retainer preference, 66.7% choose coaxial group and 33.3% liked the V-loop retainers, however with no statistical difference. 

**Table 4:** Questionnaire results for the two types of retainers after twelve months.

Questions	V-Loop n (%)	Coaxial n (%)	p -value
Comfort (1)	2 (13.3)	13 (86.7)	0.005
Discomfort (0)	13 (86.7)	2 (13.3)	
Ease of cleaning (1)	9 (60.0)	6 (40.0)	0.439
Difficulty in cleaning (0)	6 (40.0)	9 (60.0)	
Dental floss guide required (1)	7 (46.7)	8 (53.3)	0.796
Dental floss guide not required (0)	8 (53.3)	7 (46.7)	
Preferred (1)	5 (33.3)	10 (66.7)	0.197
Not preferred (0)	10 (66.7)	5 (33.3)	

When evaluating hygiene efficiency on the right and left sides, the PI was more evident on the right side (*p*<0.001). However, GI presented a statistically significant difference only for the V-loop retainers in the lingual surface (*p*<0.016). The CI showed no difference between both sides. 

Regarding bonding rate failure, V-loop retainers presented 11 events (9 had partial debonding failure and 2 were completely debonded). Coaxial retainers showed only one partial bonding failure (*p*<0,002) (Table 5).

**Table 5:** Bonding failure rate for each type of retainer.

	V-Loop n (%)	Coaxial n (%)	p Valor
Bonding failure	11 (73.3)	1 (6.6)	p > 0.002
No bonding failure	4 (26.6)	14 (93.3)	

## DISCUSSION

Periodontal health is fundamental for the oral environment and studies show that when using fixed orthodontic retainers, the patient is subject to an accumulation of calculus, gingival bleeding and possibly more severe consequences to the periodontium, but that generally do not lead to deleterious damage.^10^ ^-^ ^14^ ^,^ ^17^ ^,^ ^18^ ^,^ ^23^ ^-^ ^26^ On the other hand, stability after orthodontic treatment is more controlled with the use of lower fixed retainers for most patients with acceptable periodontal health.^1^ ^-^ ^5^ ^,^ ^8^ ^,^ ^23^ ^,^ ^27^ ^-^ ^29^

The results indicate the rejection of the null hypothesis and show a statistically significant difference between the retainers regarding the PI, which was slightly higher for the V-loop retainers, for all evaluated surfaces (Table 1). However, GI and CI did not differ between groups, presenting only a slightly greater accumulation of plaque and a slight gingival alteration in the V-loop retainers (Tables 2 and 3). For this reason, the retainers can be considered clinically similar in terms of the periodontal aspects.

A previous study that considered the retainers bonded to all the teeth concluded that these retainers show a greater accumulation of plaque and calculus than those bonded only to the canines.^10^ Although bonding on all teeth implies greater plaque and calculus accumulation, another study conducted a systematic review to evaluate the stability after orthodontic treatment comparing the retainers bonded to all the teeth and those bonded only to the canines. The authors concluded that stability was higher for the retainers in which all lower anterior teeth were bonded.^23^

Buzatta et al.^14^ conducted a systematic review with the intention of evaluating the periodontal parameters of fixed retainers that do not prevent the access of dental floss, compared to those that do. Clinical trials and cross-sectional studies that compare the two types of retainers were included. Four studies met the inclusion criteria, and all presented risk of moderate bias. Two of these studies found a statistically significant difference in gingival indexes, while the other two did not report any difference. They concluded that there is no evidence to either support or refute the association between the type of retainer and gingival health, but rather a higher flossing frequency may be a determining factor for periodontal health during the use of these retainers.

On the other hand, Lukianchuki et al^24^ compared the multi-stranded wire retainers and the modified wave-type retainers, and observed that the straight wire retainers presented better results in relation to the modified ones, when it comes to periodontal parameters, comfort, and preference. Therefore, they considered that the type of retainer interferes with these parameters. 

To ensure stability after orthodontic treatment, the orthodontist should bond the retainer on all lingual surfaces of the six lower anterior teeth. However, this condition leads to a greater accumulation of plaque and calculus in those teeth. It is important to highlight that lack of hygiene and flossing is one of the main factors for the change in periodontal health. 

In the present study, patient variable was eliminated, since the patients used both types of retainers. V-loop retainers were considered by the patients to be the most appropriate for hygiene due to its design, with no impediment to flossing. However, it seems that the greater accumulation of plaque occurred due to the greater contact area between wire and lingual surface of the lower anterior teeth and the loops of the retainers, creating a mechanical barrier for the brush bristles. 

In the present study, the patients preferred the comfort of the coaxial retainers and the ease of performing hygiene with slight advantage for V-loop retainers, but in general the coaxial retainers were preferred, but without significant differences (Table 4).

Patients reported that the V-loop retainers bothered their tongue, almost like having a “spur”. While the coaxial ones were easier to adapt to, for not having an overjet and allowing more space for the tongue. However, V-loop retainers could be used in cases treating anterior open bite, by taking advantage of this supposed discomfort.

The sample in this study included right-handed individuals who brushed better on the left side. This curious fact was confirmed by the PI altered for the right side, corroborating those studies seen in the literature.^30^ ^,^ ^31^ It is worth mentioning that the CI and GI (except for the V-loop retainers on the lingual surface) did not show any statistical difference regarding hygiene, when comparing the right and left quadrants of the lower anterior teeth. Although it was not the main target of the present study, it seems interesting to warn right-handed patients regarding the care when brushing their right lower teeth, and theoretically the inverse for left-handed patients.^31^

Another secondary outcome was regarding the higher prevalence of bonding failure in V-loop retainers, compared to coaxial retainers, corroborating previous studies.^32^ V-loop retainer has an extension towards the lingual, to some extent it creates a plateau that receives more vertical forces when chewing. It should also be checked for possible partial debonding and the consequences this might have to the periodontium. Furthermore, it is possible to say that bonding failure may compromise stability, as the patient may not promptly identify such failure, and teeth may move. Besides that, periodic revision is indicated for all types of the retainers, to prevent complications^13^ ^,^ ^32^ (Table 5).

As a limitation of this study, one may consider that six months of observation may not be sufficient to understand the impact of retainers in the periodontium. However, in a classical study, Löe^33^ investigated the evolution of gingivitis formation and how the periodontium behaves with time. A formation of gross accumulations of soft debris and subsequent marginal gingivitis were observed. The time taken to develop gingivitis ranged from 10 to 21 days. Therefore, we considered this study to establish the minimum exposure time of retainers in the oral environment. This experimental protocol was based on previous studies^17^ ^,^ ^18^ demonstrating that six months is enough time to check for such periodontal indexes evaluation comparing retainers type.

The results of the present study indicate that the coaxial retainers behaved slightly better on the periodontal condition in short time. However, as those differences were clinically small, V-loop retainers may be a viable alternative. V-loop retainer could be indicated for those patients who choose the ease of flossing to the detriment of comfort (or were treated for open bite), while the coaxial ones can be indicated for those patients who value comfort even if it makes flossing a little more difficult.

## CONCLUSION

The null hypothesis was rejected, as the coaxial retainers presented lower plaque index than the V-loop retainers (*p*<0.05). CI and GI did not differ between groups. Patients indicated the coaxial retainers as more comfortable than the V-loop retainers (*p*<0.05). Bonding rate failure was significantly small for coaxial type retainers.
